# Homozygous *TREM2* c.549del; p.(Leu184Serfs*5) variant causing Nasu‐Hakola disease in three siblings in a consanguineous Iraqi family: Case report and review of literature

**DOI:** 10.1002/mgg3.2476

**Published:** 2024-06-18

**Authors:** Naser Gilani, Fatemeh Bitarafan, Mehmet Ozaslan, Sarah Åsheim, Morteza Heidari, Masoud Garshasbi

**Affiliations:** ^1^ Department of Biology Gaziantep University Gaziantep Turkey; ^2^ Farabi Molecular Laboratory Irbil Iraq; ^3^ Department of Medical Genetics Oslo University Hospital and University of Oslo Oslo Norway; ^4^ Myelin Disorders Clinic, Department of Pediatric Neurology, Children's Medical Center, Pediatrics Center of Excellence Tehran University of Medical Sciences Tehran Iran; ^5^ Department of Medical Genetics, Faculty of Medical Sciences Tarbiat Modares University Tehran Iran

**Keywords:** disease‐associated microglia (DAM), genetic variations, Nasu‐Hakola disease (NHD), neurodegenerative disorders, TREM2, whole‐exome sequencing (WES)

## Abstract

**Background:**

The Triggering Receptor Expressed on Myeloid Cells 2 protein (TREM2) plays a crucial role in various biological processes, including osteoclast differentiation, and disease‐associated microglia (DAM) activation to regulate neuroinflammation, and phagocytosis in the brain. Genetic variations in *TREM2* are implicated in neurodegenerative disorders, such as Nasu‐hakola disease (NHD), characterized by bone lesions, neuropsychiatric disorders, and early‐onset dementia.

**Methods:**

We studied 3 siblings with suspected NHD. Whole‐exome sequencing was conducted on the proband to identify the possible genetic cause(s) and by Sanger sequencing to validate the identified variants in the two other affected siblings, a healthy sister, and the parents.

**Results:**

We identified a novel homozygous deletion (c.549del; p.(Leu184Serfs*5)) in *TREM2*. Our literature review reveals 16 *TREM2* mutations causing early‐onset dementia and bone lesions.

**Conclusion:**

These findings, alongside previous research, elucidate the clinical spectrum of TREM2‐related diseases, aiding accurate diagnosis and patient care. This knowledge is vital for understanding TREM2‐dependent DAM and its involvement in the pathogenesis of neurodevelopmental disorders which can help to develop targeted therapies and improve outcomes for TREM2‐affected individuals.

## Graphical abstract

Selective brain MRI sequences “A–C” and brain CT scan “D” of the proband: (A) Fluid Attenuated inversion recovery (FLAIR) sequence shows atrophy of basal ganglia (caudate and putamen) and deep white matter periventricular hyperintensities. (B) An axial T2‐ T2‐weighted image (T2WI) with cortical atrophy and ex‐vacuo ventriculomegaly. (C) A sagittal T2‐weighted image shows the thinning of the corpus callosum. (D) Brain CT scan shows basal ganglia calcifications. (E) The Pedigree of the investigated family, and (F) Sanger sequencing chromatograms showing nucleotide sequences of *TREM2* in the regions of c.549del which is found in the family.

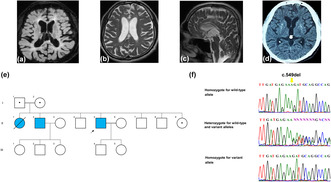

## 1 INTRODUCTION

Microglial dysfunction is a common occurrence of various neurodegenerative conditions, notably Alzheimer's disease (AD). Microglia, being highly adaptable immune cells, undergo structural changes in response to alterations in their environment. Under normal conditions, microglia maintain a resting state characterized by a ramified structure, with compact cell bodies and elongated branches, facilitating constant surveillance of their surroundings. However, exposure to stressors such as trauma or pathogens prompts their transition into an activated state, marked by thicker, retracted processes and migration toward sites of pathology (Xu et al., 2022).

Accumulation of amyloid β‐protein (Aβ) plaques and neurofibrillary tangles (NFTs) is marked in progressive neurodegenerative disorders. AD patients often exhibit widespread microglial activation across the brain, accompanied by an increased release of pro‐inflammatory cytokines, leading to a state of neuroinflammation. Unlike the typical ramified phenotype observed in healthy individuals, the presence of Aβ plaques and hyperphosphorylated tau in AD prompts microglial transformation into highly phagocytic, amoeboid cells. These transformed microglia secrete pro‐inflammatory molecules, further contributing to neuroinflammation (Xu et al., 2022).

As individuals age, the phagocytic capacity of microglia declines, resulting in inefficient clearance of pathogenic proteins like Aβ plaques and hyperphosphorylated tau. This impaired clearance exacerbates protein accumulation, intensifying neuroinflammation and sustaining microglial activation. Consequently, chronic neuroinflammation leads to synaptic and neuronal loss, aggravating the progression of the disease (Xu et al., 2022).

In the recent decade, the Triggering Receptor Expressed on Myeloid Cells 2 (TREM2) is known to be a key switch in microglial physiology in disease, regulating microglial activation, phagocytosis, and inflammatory responses. Dysregulation of TREM2 function has been implicated in various neurodegenerative diseases, highlighting its significance in maintaining microglial homeostasis and responding to pathological stimuli (Xu et al., 2022).

The expression of this receptor is prevalent in various myeloid cells, including microglia, dendritic cells, macrophages, and osteoclasts. TREM2 is distributed throughout the central nervous system (CNS), with notable abundance in white matter. Furthermore, within the CNS, TREM2 is predominantly expressed in microglia (Bonham et al., 2017; Guerreiro, Wojtas, et al., 2013; Karch & Goate, 2015; Kulkarni et al., 2021; Xing et al., 2015).

Homozygous *TREM2* variants were initially detected in patients with Nasu‐Hakola disease (NHD) also known as polycystic lipomembranous osteodysplasia with sclerosing leukoencephalopathy 2 (PLOSL2; OMIM 618193). NHD is a rare autosomal recessive disorder characterized by early‐onset progressive dementia and bone abnormalities, including bone cysts with fractures (Paloneva et al., 2002; Xing et al., 2015). The bone disorder typically manifests in the third decade of life and is characterized by pain, swelling in the ankles and feet, and frequent bone fractures. In the fourth decade, neuropsychiatric symptoms, such as frontal lobe syndrome, emerge. These symptoms are followed by profound dementia, mobility loss, and premature death in the fifth decade (Guerreiro, Bilgic, et al., 2013; Paloneva et al., 2002; Xing et al., 2015). Subsequent studies have revealed that heterozygous *TREM2* mutations have been implicated as risk factors for multiple neurodegenerative diseases, including AD, frontotemporal dementia (FTD), Parkinson's disease (PD), and amyotrophic lateral sclerosis (ALS). *TREM2* biallelic mutations are also associated with an FTD‐like syndrome without bone abnormalities (Kleinberger et al., 2014).

The *TREM2* gene is located on chromosome 6p21.1 and encodes a 230 amino acid single‐pass transmembrane immunoreceptor. The TREM2 protein is characterized by three distinct regions: an N‐terminal matured extracellular domain (ectodomain; ECD) spanning residues 19–174, a membrane‐spanning region covering residues 175–195, and a C‐terminal cytosolic tail encompassing residues 196–230. The first 18 amino acids serve as a signaling peptide for the protein. The ECD domain consists of three crucial complementarity‐determining regions (CDR loops). These regulatory loops play a significant role in ligand interaction. Recent investigations have highlighted the adverse impact of mutations on these specific regions (Swain et al., 2023).

To date, over 70 TREM2 variants have been documented across a spectrum of neurodegenerative disorders, as reported in the literature, the Human Gene Mutation Database (HGMD) (https://www.hgmd.cf.ac.uk/), and the Alzforum database (https://www.alzforum.org/). Among these variants, we identified a compound heterozygous mutation and 15 homozygous mutations in patients presenting with early‐onset progressive dementia and bone abnormalities (Table 1). Detailed phenotypic descriptions of these patients are provided in Table S1. These mutations included 7 missenses listed as c.113A>G; p.(Y38C), c.150G>T; p.(W50C), c.197C>T; p.(T66M), c.257A>T; p.(D86V), c377T>G; p.(V126G), c.401A>G; p.(D134G), located in the ECD of the protein, and c.558G>T; p.(K186N), located in the transmembrane domain of TREM2.

**TABLE 1:** Disease‐causing variants associated with presenile dementia with bone abnormalities.

	Position GRCh37/hg19	Exon/intron no.	HGMD accession	dbSNP	Variant class (HGMD)	Nucleotide change (cDNA position)	Amino acid change	Type of mutation	Reported phenotype	Reference
1	Chr6:41130781	exon 1	CM034622	rs386834143	DM	c.40G>T	p.(Glu14stop)	Nonsense	PLOSL	Paloneva et al. (2003)
2	Chr6:41129295	exon 2	CM033823	rs104894002	DM	c.97C>T	p.(Gln33stop)	Nonsense	PLOSL, FTD without any bone cysts or other bone phenotype, and AD	Guerreiro, Wojtas, et al. (2013); Guerreiro, Lohmann, et al. (2013); Borroni et al. (2014); Klünemann et al. (2005); Soragna et al. (2003); Montalbetti et al. (2005); Ghezzi et al. (2017)
3	Chr6:41129279	exon 2	CM130450	rs797044603	DM	c.113A>G	p.(Tyr38Cys)	Missense	PLOSL, FTD without any bone cysts, and AD	Guerreiro, Wojtas, et al. (2013); Guerreiro, Lohmann, et al. (2013); Samanci et al. (2021); Köseoğlu et al. (2018)
4	Chr6:41129260	exon 2	CM022074	rs104894001	DM	c.132G>A	p.(Trp44stop)	Nonsense	PLOSL	Paloneva et al. (2002)
5	Chr6:41129242	exon 2	CM1721742	NA	DM	c.150G>T	p.(Trp50Cys)	Missense	PLOSL	Dardiotis et al. (2017)
6	Chr6:41129195	exon 2	CM130449	rs201258663	DM	c.197C>T	p.(Thr66Met)	Missense	PLOSL, and FTD without any bone cysts or other bone phenotype	Guerreiro, Wojtas, et al. (2013); Guerreiro, Lohmann, et al. (2013); Borroni et al. (2014); Samanci et al. (2021); Sasaki et al. (2015); Le Ber et al. (2014); Thelen et al. (2014)
7	Chr6:41129193	exon 2	NA	NA	NA	c.199delC	p.(His67Thrfs*9)	Frameshift Deletion	PLOSL^a^	Buthut et al. (2023)
8	Chr6:41129159	exon 2	CM022075	rs104893998	DM	c.233G>A	p.(Trp78Stop)	Nonsense	PLOSL	Paloneva et al. (2002)
9	Chr6:41129123	exon 2	CD058316	rs386834140	DM	c.269delG	p.(Gly90Valfs*99)	Frameshift Deletion	PLOSL	Klünemann et al. (2005)
10	Chr6:41129079	exon 2	CD058317	rs386834141	DM	c.313delG	p.(Ala105Argfs*84)	Frameshift Deletion	PLOSL	Klünemann et al. (2005)
11	Chr6:41129135	exon 2	CM139681	NA	DM	c.257A>T	p.(Asp86Val)	Missense	PLOSL, and FTD	Samanci et al. (2021)
12	Chr6:41129015	exon 2	CM058315	rs121908402	DM	c.377T>G	p.(Val126Gly)	Missense	PLOSL	Klünemann et al. (2005)
13	chr6:41129000	intron 2	CS201910	NA	NA	c.391+1G>A	‐	Splice site	FTD with mild radiological bone involvement	Le Ber et al. (2014)
14	Chr6:41127611	exon 3	CM022076	rs28939079	DM	c.401A>G	p.(Asp134Gly)	Missense	PLOSL	Paloneva et al. (2002)
15	Chr6:41127528	intron 3	CS022111	rs386834144	DM	c.482+2T>C	‐	Splice site	PLOSL	Paloneva et al. (2002); Numasawa et al. (2011)
16	Chr6:41126738	exon 4	NA	NA	NA	c.549delA	p.(Leu184Serfs*5)	Frameshift Deletion	PLOSL	This study
17	Chr6:41126729	exon 4	CM022077	rs28937876	DM	c.558G>T	p.(Lys186Asn)	Missense	PLOSL	Paloneva et al. (2002)

Abbreviations: DM, disease causing mutation; NR, not available.

^a^ This variant is identified in compound heterozygosity with c.313delG;p.Ala105Argfs*84.

We also observed four nonsense mutations: c.40G>T; p.(E14X), resulting in a truncated protein at codon 14 within the signal peptide of the TREM2 protein; c.97C>T; p.(Q33X), c.132G>A; p.(W44X), and c.233G>A; p.(W78X), which are predicted to lead to the generation of truncated proteins lacking the transmembrane and cytoplasmic domains.

Additionally, two splicing site mutations, c.482+2T>C and c.391+1G>A, were identified, producing an aberrant protein.

Furthermore, three small deletions involving single‐nucleotide deletions at positions 199, 269, and 313 caused frameshifts, producing truncated polypeptides due to premature translation termination. In this study, we also report a novel homozygous deletion, c.549del; p.(Leu184Serfs*5), in the *TREM2* gene (NM_018965) in three siblings from an Iraqi consanguineous family who exhibited phenotypic features consistent with NHD.

## 2 MATERIALS AND METHODS

### 2.1 Ethical consideration

This research has been conducted ethically in accordance with the World Medical Association Declaration of Helsinki; informed consent was obtained and the study was approved by the local medical ethics committee of DeNA laboratory, Tehran, Iran.

### 2.2 Clinical findings

In this study, we reported three siblings who presented with NHD in a consanguineous Iraqi family.

The proband (II‐5), a 35‐year‐old man, presented with skeletal pain, joint swellings, bone cysts, frequent bone fractures, personality changes, early onset dementia, tremors, inappropriate and risky behaviors, apathy, bradykinesia, and generalized tonic–clonic seizures. The psychological problems began at the age of 12, whereas the skeletal disorder manifested in the third decade of his life. Currently, he is undergoing antiepileptic medication treatment.

**FIGURE 1:**
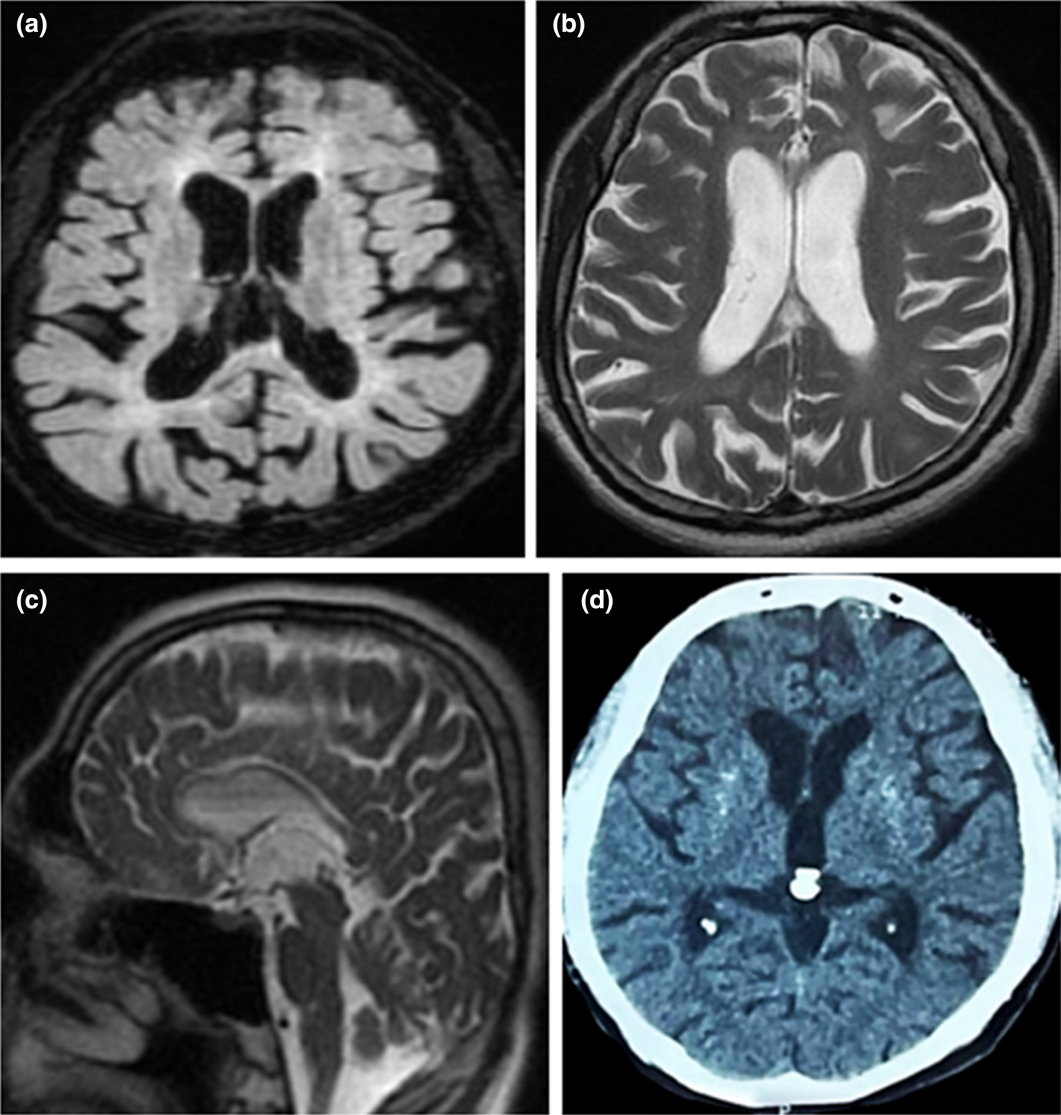
Selective brain MRI sequences “a–c” and brain CT scan “d” of the proband: (a) Fluid Attenuated inversion recovery (FLAIR) sequence shows atrophy of basal ganglia (caudate and putamen) and deep white matter periventricular hyperintensities. (b) An axial T2‐ T2‐weighted image (T2WI) with cortical atrophy and ex‐vacuo ventriculomegaly. (c) A sagittal T2‐weighted image shows the thinning of the corpus callosum. (d) Brain CT scan shows basal ganglia calcifications.

A comprehensive neuroimaging evaluation was conducted to investigate the underlying brain's structural changes in the proband. On neuroimaging, the Fluid Attenuated Inversion Recovery (FLAIR) sequence revealed marked atrophy of the basal ganglia, specifically affecting the caudate and putamen regions. In addition, deep white matter periventricular hyperintensities were observed, suggesting underlying degenerative changes. An axial T2‐weighted image (T2WI) demonstrated cortical atrophy and ex‐vacuo ventriculomegaly. Further evaluation with a sagittal T2‐weighted image revealed thinning of the corpus callosum. The Brain CT scan displayed the presence of basal ganglia calcifications (Figure 1). The combination of atrophy in the basal ganglia and deep white matter, cortical atrophy, ventriculomegaly, thinning of the corpus callosum, and basal ganglia calcifications raises concerns about an underlying neurodegenerative disorder.

The affected brother (II‐2), who is 40 years old, also presents with a combination of physical and mental symptoms indicative of a severe medical condition. He exhibits skeletal pain, joint swellings and bone cysts, along with a history of frequent bone fractures in his 30s, suggesting underlying skeletal abnormalities. Additionally, notable neurological symptoms are evident, including personality changes, early‐onset dementia, tremors, poor judgment, bradykinesia, and generalized tonic–clonic seizures which collectively point toward a progressive neurodegenerative disorder. These symptoms encompass a range of physical and cognitive impairments, reflecting the complex and debilitating nature of the condition affecting him. Unfortunately, neuroimaging or other diagnostic tests to further investigate his condition are unavailable. The onset of psychological problems occurred at the age of 10. Currently, he is receiving antiepileptic therapy.

The affected sister (II‐1) is characterized by a diverse array of symptoms, reflecting a complex medical condition. She presents with skeletal pain, joint swellings, bone cysts, and a history of frequent bone fractures in the third decade of her life, indicating significant skeletal involvement. Alongside these physical manifestations, notable neurological symptoms are evident, including personality changes, early‐onset dementia, and tremors. At age 10, the first signs of psychological difficulties emerged. Sadly, she passed away at the age of 41 after mobility loss, highlighting the severity and profound impact of her condition. No neuroimaging evaluation or other medical tests were conducted to investigate her disease.

Their 62‐year‐old mother (I‐2) exhibits personality changes, inappropriate behavior, aggressiveness, depression, and tremors. Despite these challenges, she manages to care for her affected children. The onset of her psychological problems began at the age of 37, though no evaluations via neuroimaging or other medical tests were carried out to investigate her condition. She is currently receiving treatment for depression. She has not experienced joint swellings and frequent bone fractures. Their 65‐year‐old father (I‐1) had no history of movement disorders, seizures, early‐onset dementia, or any other neuropsychiatric symptoms.

### 2.3 Genetic findings

**FIGURE 2:**
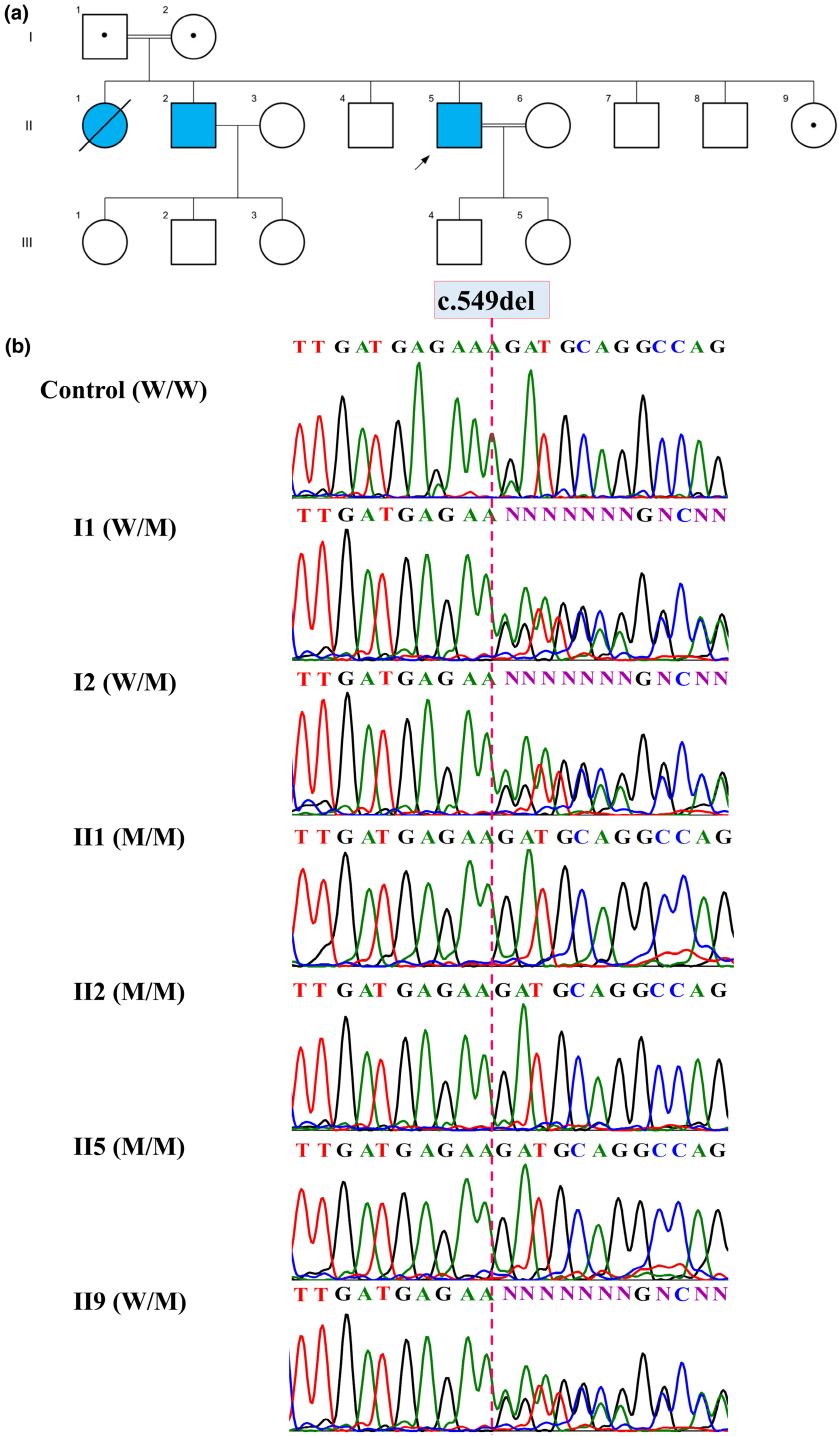
The Pedigree of the investigated family, and Sanger sequencing chromatograms: (a) The Pedigree of the investigated family, and in this pedigree, an arrow indicates the proband. Blue symbol: affected and homozygous for the variant; dotted symbols: carriers for the variant; squares: males; circles: females; parallel lines: consanguineous marriage. (b) Sanger sequencing chromatograms showing nucleotide sequences of *TREM2* in the regions of c.549del found in the family. W/W is homozygous for Wild‐type allele, W/M is heterozygote, and M/M is homozygous for c.549del.

Peripheral blood samples were collected from three affected patients (II1, II2, II5), a healthy sister (II9), and their parents (I1, I2) for genomic DNA extraction. Genomic DNA extraction was performed using the High Pure PCR template preparation kit (Roche; Product No. 11814770001). Whole‐exome sequencing (WES) was conducted on the proband (II5; Figure 2a).

To capture approximately 60 Mb of the Human Exome from fragmented genomic DNA, the Agilent SureSelect Human All Exon V6 kit was utilized. The generated library was sequenced on an Illumina Hiseq 4000 platform to achieve an average coverage depth of 100. Over 97% of the targeted bases were covered with a depth greater than 10. An end‐to‐end in‐house bioinformatics pipeline involving base calling, alignment of reads to GRCh37/hg19 genome assembly, primary filtering out of low‐quality reads and probable artifacts, and subsequent annotation of variants, was used.

Using the Burrows‐Wheeler Aligner (http://bio‐bwa.sourceforge.net/) reads were mapped to the reference human genome. Single‐nucleotide variants (SNVs) and micro insertions‐deletions (indels) were called using SAMtools (http://samtools.sourceforge.net/) with filtered variants having a mapping quality score of greater 20 and were annotated using ANNOVAR (http://www.openbioinformatics.org/annovar/). The analysis focused on coding exons and the flanking ±20 intronic bases. Disease‐causing variants reported in Human Gene Mutation Database (HGMD) and ClinVar, along with variants having a minor allele frequency (MAF) of less than 1% in publicly available mutation and polymorphism databases like the 1000 genome project, ExAC (Exome Aggregation Consortium), ESP (Exon Sequencing Projects), gnomAD, and Iranome database, were considered.

After several filtering steps, WES analysis revealed a novel homozygote deletion variant in the *TREM2* gene, c.549del; p.(Leu184Serfs*5), compatible with the diagnosis of NDH. To validate the variant's segregation within the family, Sanger sequencing on the three affected siblings (II1, II2, II5), a healthy sibling (II9), and their parents (I1, I2) were performed (primers and conditions are available upon request). DNA sequencing was performed on ABI 3130 with the ABI PRISM BigDye Terminator v. 3.1 sequencing kit (Applied Biosystems, USA). Sequencing chromatograms were analyzed using Codon Code Aligner software version 8.0.2 (CodonCode Corp, USA). The affected siblings were found to be homozygous for the mutant allele, whereas the parents and healthy sister were heterozygous (Figure 2b).

## 3 DISCUSSION

Over the past decade, research has shed light on a phenotypic and functionally distinct subset of microglia known as disease‐associated microglia (DAM) or “neurodegenerative” phenotype, which is closely linked with neurodegenerative disorders including AD, FTD, ALS, and aging. In the recent decade, TREM2 has been known to be a key switch in microglial physiology in disease.

Research findings indicate that in individuals without health complications, microglia usually adopt a ramified morphology and express a wide range of marker genes associated with homeostatic microglia. However, during the initial stages of AD before the formation of Aβ plaques and NFTs, microglia undergo a transition to a highly proliferative state, accompanied by an increase in the production of various chemokines and inflammatory genes. As the accumulation of Aβ plaques progresses, some microglia downregulate the expression of marker genes associated with normal microglial function, transitioning into stage 1 DAM, and localizing near the Aβ plaques. With continued Aβ and NFT deposition, stage 1 DAM gradually evolves into fully activated stage 2 DAM via pathways dependent on TREM2. Research indicates that genetic deletion of the TREM2 gene impairs the transition from DAM1 to DAM2 (Sobue et al., 2021; Xu et al., 2022). The necessity of TREM2 for the complete activation of the DAM program in mice suggests that the functionality of DAMs could potentially serve as a protective mechanism against disease development. DAM2 demonstrates high phagocytic activity and actively internalizes Aβ plaques by upregulating genes involved in phagocytosis and lipid metabolism (Deczkowska et al., 2018).

Research has verified the existence of TREM2‐dependent DAM in AD patients who carried *TREM2* variants, as well as in 5xFAD mice lacking the *TREM2* gene, exhibiting decreased expression of DAM signature genes. However, the precise molecular mechanisms involved in the transition from normal microglia to DAMs remain unclear and require further investigation (Deczkowska et al., 2018).

Studies have demonstrated that overexpression of TREM2 upregulates genes associated with microglial phagocytosis, anti‐inflammatory responses, and microglial survival in 5xFAD mice. This leads to an elevation in the production of neuroprotective anti‐inflammatory cytokines and a reduction in the production of neurotoxic pro‐inflammatory cytokines. Furthermore, mutations causing a loss of function in genes upregulated by the DAM are linked to a higher susceptibility to AD, conversely, mutations resulting in a gain of function in genes downregulated by the DAM increase susceptibility to AD. These findings suggest that the DAM program constitutes a defensive innate immune response against disease pathology. In a recent study, it has been shown that TREM2 has emerged as a promising therapeutic target for AD treatment since TREM2 overexpression rescued cognitive impairments in mice models by inhibiting microglia‐mediated neuroinflammation (Deczkowska et al., 2018).

The comprehensive understanding of TREM2‐dependent DAM and its involvement in the pathogenesis of neurodegenerative disorders remains complex. Although it's generally agreed upon that DAM plays a protective role in slowing down AD progression, recent studies have identified distinct subsets within DAM, categorized as a dual role for TREM2 in AD pathogenesis; “pro‐inflammatory” and “anti‐inflammatory.” Analysis of the upstream regulators of major DAM markers reveals the contribution of both pro‐ and anti‐inflammatory signaling pathways to DAM formation. This complexity indicates that TREM2‐dependent DAM may have diverse roles. Thus, the regulatory network governing TREM2‐dependent DAM involves a combination of pro‐ and anti‐inflammatory pathways, underscoring its intricate nature (Xue & Du, 2021; Yao et al., 2019).

*TREM2* variants have been reported in a diverse array of neurodegenerative disorders, including NHD, FTD, AD, PD, semantic variant of primary progressive aphasia (svPPA), and ALS (Bonham et al., 2017; Dardiotis et al., 2017; Kleinberger et al., 2014).

Studies showed that *TREM2* variants linked to NHD are usually situated in the buried region of the TREM2 protein, whereas the residues associated with the AD are typically found on the surface of the protein. These findings suggest that alterations linked to NHD disrupt the structure of the TREM2 protein through mechanisms such as misfolding, truncation, or unfolding (Swain et al., 2023). Thus far, a compound heterozygous mutation and 15 homozygous mutations have been documented, comprising 7 missense, 4 nonsense, 2 splicing site mutations, and 3 small deletions in individuals affected by presenile dementia with bone abnormalities. Some of these variants have also been identified in other neurodegenerative disorders, including FTD and AD (Borroni et al., 2014; Guerreiro, Lohmann, et al., 2013; Guerreiro, Wojtas, et al., 2013; Samanci et al., 2021; Sasaki et al., 2015). In this study, we also report a novel homozygous deletion, c.549del; p.(Leu184Serfs*5), in the *TREM2* gene in three siblings from an Iraqi consanguineous family who exhibited phenotypic features consistent with NHD.

Missense mutations occurring at conserved or crucial sites often disrupt interactions under native conditions, leading to decreased protein stability, solubility, and signaling (Swain et al., 2023). Most of the missense mutations related to NHD, including c.113A>G; p.(Y38C), c.150G > T; p.(W50C), c.197C > T; p.(T66M), c.257A>T; p.(D86V), c.377T>G; p.(V126G), and c.401A>G; p.(D134G), are located in the ECD of the TREM2 protein, crucial for ligand interaction and signaling (Swain et al., 2023). Additionally, one missense mutation is situated in the transmembrane domain of TREM2: c.558 G>T; p.(K186N) (Paloneva et al., 2002).

The c.113A>G; p.(Y38C) missense mutation, which is located in the first CDR loop of the ECD of the protein, impacts the post‐translational process of TREM2, as well as the binding of ligands, also leading to a reduction in the phagocytosis process mediated by TREM2 (Kleinberger et al., 2014; Kober et al., 2016; Park et al., 2015; Sirkis et al., 2017; Swain et al., 2023). The c.113A>G; p.(Y38C) was reported in a Turkish family with multiple affected individuals with NHD. Köseoğlu et al. (2018) investigated a 35‐year‐old woman with cognitive deficits progressing for 2 years, personality and behavior changes, and a medical history of foot and leg pain with repetitive fractures during the preceding 10 years. Diffuse signal changes in the subcortical frontal and parietal white matter, along with global brain atrophy, a thin corpus callosum, mild basal ganglia calcification, a bilateral hypometabolic pattern involving basal nuclei, temporal cortex, and anterior cingulate gyrus were detected. X‐ray imaging of both talus bones showed osteoporotic and cystic lesions. The 41‐year‐old patient's uncle also exhibited personality and behavioral changes that had been ongoing for a year. CT examination revealed cerebral atrophy and mild basal ganglia calcification. Brain MRI showed diffuse signal alterations in the subcortical frontal and parietal white matter, along with global brain atrophy, a diffusely thinned corpus callosum, and reduced anterior cingulate gyrus volume. CT exhibited bilateral hypometabolism in the anterior cingulate gyrus, basal nuclei, and parietotemporal cortex. X‐ray and CT images of bilateral ankles indicated a reduction in trabecular structure and multiple lytic lesions with sclerotic rims in the talus (Köseoğlu et al., 2018).

The c.113A>G; p.(Y38C) mutation was previously reported in a Turkish family presenting with a behavioral FTD‐like syndrome without bone cysts (Guerreiro, Lohmann, et al., 2013). In 2021, Samanci et al. (2021) reported another patient with the homozygote c.113A>G, p.(Y38C) variant, who exhibited behavioral changes along with cognitive decline. Her medical history did not show any bone cysts or fractures (Samanci et al., 2021). Additionally, in a study by Guerreiro, Wojtas, et al. (2013), the heterozygous p.(Y38C) mutation was reported in AD patients but not in the controls. (Guerreiro, Wojtas, et al. 2013)

W50 is predicted as a highly conserved residue in the TREM2 protein family. It is located in the core of the TREM2 protein and plays a crucial role in upholding the structural integrity of the protein. The p.(W50C) mutation was predicted to be deleterious by two in silico prediction software tools, Polyphen‐2 and SIFT (Dardiotis et al., 2017; Swain et al., 2023). Dardiotis et al. (2017) identified a homozygous missense variant, c.150G>T; p.(W50C), in a 33‐year‐old Greek female with a phenotype suggestive of NHD. This patient had a 3‐year history of progressive cognitive decline. At the age of 17, a brain CT scan was conducted due to episodes of acute, short‐lasting, neuralgic pain in the right occipital area, revealing calcifications in the basal ganglia. She experienced her first fracture in her left wrist at the age of 28. At the age of 30, her personality changed, and she gradually developed behavioral alterations, indicating frontal lobe dysfunction. These alterations included emotional instability, euphoria, lack of insight, bulimia, perseveration, and stereotyped behaviors. Additionally, she exhibited increased speech velocity, gait instability, and urinary incontinence. Brain MRI revealed diffuse symmetrical hyperintensities in the white matter and very low signal intensities of the basal ganglia on T2‐weighted images, along with brain atrophy and a thin corpus callosum. CT showed cerebellar atrophy, diffuse low‐density areas in the brain white matter, and the presence of subependymal and basal ganglia calcifications. A radiographic examination of the bones revealed multiple diffuse cystic and osteosclerotic lesions (Dardiotis et al., 2017).

Residue 66 is situated within the second CDR loop. Variations in these CDR loops are associated with the disruption of ligand interactions. Recent studies have highlighted the detrimental impact of mutations affecting these specific regions. According to studies, it has been observed that amino acid 66 is deeply located within the folded structure of the TREM2 protein. This observation has led to the hypothesis that the p.(T66M) mutation interferes with the proper folding of the protein (Kober et al., 2016; Kulkarni et al., 2021). In 2015, Sasaki et al. (2015) investigated the brain autopsy of a Japanese patient with NHD who had a homozygous missense mutation, c.197C> T; p.(T66M), causing a polarity shift. The patient passed away at the age of 41. Neurological symptoms, including memory loss, first appeared when she was 35 (Sasaki et al. 2015). At the age of 40, she developed extrapyramidal signs, epilepsy, and dementia. Additionally, bone fractures and lipomembranous lesions of the bone were observed. The brain autopsy revealed severe brain atrophy with diffuse loss of myelin and axons in the white matter, particularly accentuated in the frontal lobe. Her elder sister had also died of NHD at the age of 37 (Sasaki et al., 2015). Le Ber et al. (2014), described an Italian sibling with a homozygous *TREM2* mutation, p.(T66M), associated with the early onset Behavioral variant of Frontotemporal dementia (bvFTD). The proband did not have severe bone disorders, whereas the other sibling had fractures in the ankle and foot due to trauma but no other bone phenotype (Le Ber et al. 2014).

The homozygous c.197C>T; p.(T66M) variant is also associated with FTD‐like syndrome without any history of bone cysts or fractures (Guerreiro, Lohmann, et al., 2013; Samanci et al., 2021). Additionally, literature has linked the heterozygous p.(T66M) variant to other neurodegenerative disorders such as FTD and AD (Borroni et al., 2014; Guerreiro, Wojtas, et al., 2013; Thelen et al., 2014).

D86 is predicted as a highly conserved Residue in the TREM2 protein family. Studies revealed that p.(D86V) mutation disrupts protein maturation leading to decreased cell‐surface expression (Sirkis et al., 2017; Swain et al., 2023). In 2021, Samanci et al. (2021) conducted a study focusing on 14 patients who were presenting behavioral changes with cognitive decline. Thinning of the Corpus callosum, White matter involvement, and symmetrical global atrophy of the brain, mainly affecting frontoparietal and lateral temporal regions, were observed in all cases. Within this group, 5 patients were identified as homozygous for the c.257A>T; p.(D86V) missense variant. The onset of symptoms varied between 33 to 38 years of age. These individuals all experienced parkinsonism, and 3 of them also presented epileptic seizures. Additionally, CT scans revealed calcification in 4 patients. Interestingly, among the patients, there were skeletal findings. Specifically, one patient exhibited bilateral talus bone cysts without any fractures, another had a history of fractures but no bone cysts, and a third patient showed both bone cysts and a history of fractures (Samanci et al., 2021). Compound heterozygosity of p.(D86V) with p.(Y38C) was reported in two Turkish sisters presented with bvFTD, without any fracture history or Bone cysts (Guerreiro, Bilgic, et al., 2013).

V126 is anticipated to be in a highly conserved region within the TREM2 protein family. It is buried in the ectodomain and predicted to play a role in maintaining the hydrophobic region in the TREM2 protein. V126G exhibits reduced cell‐surface expression and an inability to undergo N‐linked glycosylation in the Golgi; however, it may potentially undergo O‐linked glycosylation through an alternative pathway (Swain et al., 2023). In 2017, Kober et al. (2016) predicted that the p.(V126G) protein variant leads to misfolding of the TREM2 protein (Kober et al. (2016). Klünemann et al. (2005) reported c.377T>G; p.(V126G) in a homozygous state in two unrelated affected individuals from Canada and the UK (Sri Lanka) who exhibited presenile dementia and bone cysts. The female patient manifested multiple cystic bone lesions and encountered two fractures, with the initial one occurring at age 29. Similarly, the male patient also displayed multiple cystic bone lesions and started experiencing bone pain at the age of 37, coinciding with his first fracture. Both individuals developed frontal lobe syndrome, marked by personality and behavioral changes, at the ages of 31 and 37 years, respectively (Klünemann et al. 2005).

The biological effect of the c.401A>G; p.(D134G) missense mutation remains unknown. Paloneva et al. (2002) identified this variant in an American patient of Slovakian origin who experienced bone abnormalities in their third decade of life, along with symptoms suggestive of frontal lobe syndrome, including euphoria and loss of social inhibitions, and progressive dementia. Additionally, the patient presented with skeletal pain, primitive reflexes, diffuse slowing in the electroencephalogram, and convulsions (Paloneva et al. 2002).

In addition to missense variants affecting the ectodomain of the TREM2 protein, a specific missense mutation in the transmembrane domain, c.558G>T; p.(K186N), predicted to result in defects in signal transduction. This mutation results in the replacement of the positively charged lysine with asparagine in the transmembrane domain of TREM2. Studies have demonstrated that this alteration disrupts the interaction with TYROBP and hinders the cell‐surface expression of TYROBP. In 2002, Paloneva et al. (2002) identified the p.(K186N) variant in a Norwegian family, where two sisters displayed a homozygous substitution, c.558G>T; p.(K186N). The affected individuals in this family shared common clinical features, including bone issues emerging in their third decade of life, frontal lobe syndrome (marked by euphoria and loss of social inhibitions), progressive dementia, and brain atrophy in their fourth to fifth decade of life. Beyond these shared symptoms, one sister presented with agnostic‐aphasic‐apraxic symptoms, whereas the other experienced convulsions (Paloneva et al., 2002; Sirkis et al., 2017).

Up to now four homozygous nonsense mutations, c.40G>T; p.(E14X), resulting in a truncated protein at codon 14 within the signal peptide of the TREM2 protein; c.97C>T; p.(Q33X), c.132G>A; p.(W44X), and c.233G>A; p.(W78X), which are expected to produce truncated proteins lacking the transmembrane and cytoplasmic domains, have also been reported in the *TREM2* gene.

The c.40G>T; p.(E14X) nonsense mutation, was reported in a PLOSL German patient. The researchers observed that TREM2 transcripts were undetectable in the stimulated human peripheral blood mononuclear cells of this German patient, likely due to the knockout nature of the mutation (Paloneva et al., 2003).

The c.97C>T; p.(Q33X) nonsense mutation has been reported in various studies involving both homozygous and heterozygous patients exhibiting different neurodegeneration disorders. This mutation is in the ECD of the TREM2 protein and is predicted to result in the generation of a truncated protein that completely lacks the transmembrane and cytoplasmic domains. This leads to a loss of function of the TREM2 protein. In addition, Q33 is located on the surface of the second Beta strand in the N‐terminal region of the protein. Mutations at this specific position result in the loss of TREM2 expression. Residue Q33 is predicted to be at the conserved region in the TREM2 protein (Dardiotis et al., 2017; Guerreiro, Lohmann, et al., 2013; Kulkarni et al., 2021; Swain et al., 2023). This variant, in a homozygous state, was first detected in two Italian siblings affected by NHD. The proband, a 46‐year‐old woman, had been in good health until the age of 23 when she began experiencing bone fractures. Subsequently, she developed insidious personality changes, loss of social inhibition and judgment, and depression with suicidal ideation starting at the age of 30. By the age of 40, she was presented with frontal signs, primitive reflexes, dyscalculia, mild apraxia, and spatial and temporal disorientation. Brain CT scans revealed diffuse cerebral atrophy with calcification in the basal ganglia. Ultimately, the disease progression led to a vegetative state due to progressive cognitive and motor dysfunctions, epileptic seizures, and cerebral ictal events. Her affected sister started experiencing progressive loss of judgment, depression, personality changes, and uninhibited attitudes at the age of 30. While she did not have bone fractures, X‐ray imaging revealed cystic bone lesions. Cognitive function deterioration, along with frontal dysfunction, dyscalculia, dysgraphia, and severe diffuse cerebral atrophy with basal ganglia calcification, was reported (Soragna et al., 2003). Among the family members, four individuals were found in a heterozygosity state, including the parents, brother, and daughter of one of the affected sisters. Despite being clinically normal, the neuropsychological tests conducted on the 46‐year‐old brother and 20‐year‐old daughter showed deficits in visuospatial memory. Furthermore, functional imaging indicated hypoperfusion in the basal ganglia region for both individuals (Montalbetti et al., 2005).

Later, researchers reported a homozygous state for the p.(Q33X) mutation in a Belgian sibling diagnosed with NHD. These affected individuals began experiencing personality and behavioral changes in their late 30s. MRI scans indicated leukoencephalopathy, cerebral atrophy, and thinning of the corpus callosum. Additionally, CT scans and SPECT imaging respectively revealed basal ganglia calcification and widespread cerebral hypoperfusion. They both suffered from multiple cystic bone lesions and experienced fractures (Klünemann et al., 2005). Ghezzi et al. (2017) documented another case of NHD in an affected individual who was homozygous for the p.(Q33X) variant. This individual exhibited personality changes, social inhibition, memory disturbances, and disorientation that began at 37 years old. Diffuse cortical atrophy, white‐matter loss, and cortical hypometabolism were reported. Multiple asymptomatic cystic bone lesions of hands and feet were observed. At about 40 years old, the patient experienced a significant decline in neurological health, resulting in complete dependence on daily activities. Additionally, she developed chronic urinary retention. The parents of this patient, who were heterozygous carriers of the p.(Q33X) variant, displayed no cognitive abnormalities at the age of 72 (Ghezzi et al., 2017).

The p.(Q33X) variant was also reported in a Turkish man, presented in a homozygous state, showing FTD‐like symptoms, but without the bone cysts (Guerreiro, Lohmann, et al., 2013). Additionally, some case–control studies, including Borroni et al. (2014), have associated heterozygous variants of the p.(Q33X) mutation with FTD. Among their cohort of 352 Italian FTD patients, three females carried heterozygous mutations that were not found in their healthy controls. Among them, two exhibited svPPA with ages of onset at 68 and 52, respectively. The third patient displayed characteristics of bvFTD with an onset age of 53 (Borroni et al. 2014). Moreover, Guerreiro, Lohmann, et al. (2013), associated heterozygous variants of the p.(Q33X) mutation with AD (Guerreiro, Lohmann, et al. 2013).

W44, an exposed hydrophobic residue, is located in the first CDR loop of the ectodomain of the TREM2 protein. Mutations in CDR loops disrupted ligand interactions (Swain et al., 2023). The c.132G>A; p.(W44X) nonsense variant, was detected in a Bolivian patient presented with bone problems in their third decade of life, frontal lobe syndrome (euphoria and loss of social inhibitions), progressive dementia, and brain atrophy in their fourth to fifth decade of life. This patient also displayed skeletal pain, agnostic‐aphasic‐apraxic symptoms, primitive reflexes, diffuse slowing in the electroencephalogram, and convulsions following neurosurgery (Paloneva et al., 2002).

Residue W78 is situated within the ectodomain and is buried. The p.(W78X) mutation leads to the expression of a truncated protein (Swain et al., 2023). The c.233G>A; p.(W78X) nonsense mutation was identified in a Swedish patient who presented with bone problems in their third decade of life, frontal lobe syndrome (euphoria and loss of social inhibitions), progressive dementia, brain atrophy in their fourth to fifth decade of life. Additionally, this patient exhibited skeletal pain, agnostic‐aphasic‐apraxic symptoms, and primitive reflexes. Interestingly, the same mutation was observed in another Swedish family with three affected individuals (Paloneva et al., 2002).

Additionally, two splicing site mutations, c.391+1G>A, and c.482+2T>C, associated with NHD were reported. These mutations lead to the production of an aberrant protein. The c.391+1G>A mutation was reported in a Chinese Han woman with bvFTD with mild radiological bone involvement. At the age of 33 years, she began experiencing behavioral changes, including loss of motivation, an inability to concentrate, poor judgments, neglect of personal hygiene, disinhibition, an unusually increased appetite, and indifference to family members and social life. She also displayed moderate aggression. Over the years, her symptoms worsened, and she developed urinary incontinence, cognitive impairment, and memory loss. At the age of 40 years, the neurological examination revealed bradykinesia, brisk reflexes, and mild ataxia. Positive bilateral Hoffman, Babinski, and Chaddock signs were observed. Brain imaging showed moderate cortical atrophy, ventricular enlargement, and thinning of the corpus callosum. Additionally, symmetrical hyperdense lesions were observed in the globus pallidus, indicating calcifications. Notable diffuse hyperintensities were also seen in the periventricular, frontal, temporal, and occipitoparietal white matter on T2 and FLAIR. The patient had no history of bone fractures in her past 40 years of life. A thorough skeletal X‐ray survey revealed mild cortical thinning and possible cystic swelling of the metaphyses of the long bones in the carpal and talus bones. This variant was identified as causing aberrant splicing (Li et al., 2020).

The splice‐donor site of intron 3 mutation, c.482+2T>C, was reported in two Italian sisters resulting in exon 3 skipping and potentially affecting the mature mRNA, leading to the production of an aberrant protein. Both sisters exhibited agnostic‐aphasic‐apraxic symptoms. Additionally, one of the patients reported skeletal pain and diffuse slowing in the electroencephalogram, whereas the other experienced convulsions and primitive reflexes (Paloneva et al., 2002). Subsequently, the c.482+2T>C variant was also documented in a Japanese family affected by NHD. Within the family, a woman and her eldest brother exhibited symptoms such as dementia, multifocal radiolucent lesions, and epileptic seizure, whereas her second elder brother presented with dementia and multifocal radiolucent lesions. The woman in her 30s started displaying a shift in her personality over time. This shift was marked by laziness, diminished ability to concentrate, a lack of insight, poor judgment, social inhibitions, apathy, and memory disturbances. Bilateral calcification of the basal ganglia, moderate hypoperfusion in the frontotemporal cortex with preservation of the cerebral blood flow in the basal ganglia, moderate atrophy, and marked dilatation of the lateral ventricle were reported. An EEG revealed sporadic spike waves in diffuse slow activities (Numasawa et al., 2011). In addition, Redaelli et al. (2018) reported this variant as a compound heterozygote accompanied by the p.(D134G) variant in a patient with FTD. This patient was characterized by behavioral changes and aphasia, displaying a very early onset and exceptionally prolonged duration of the disease. Furthermore, this patient also exhibited choreic movements but did not experience any bone fractures (Redaelli et al. 2018).

Furthermore, three small deletions involving single‐nucleotide deletions at positions 199, 269, and 313 caused frameshifts, resulting in the production of truncated polypeptides due to premature translation termination.

In 2005, Klünemann et al. (2005) reported two patients, one from Germany and the other from France (Turkey), both suffering from presenile dementia and bone cysts. These mutations were predicted to cause frameshifts, resulting in the production of truncated polypeptides due to premature translation termination. The affected woman, carrying the homozygous c.313delG; p.(Ala105Argfs*84) mutation, experienced the onset of a frontal lobe syndrome characterized by personality and behavioral changes at the age of 33 years. Additionally, she exhibited multiple cystic bone lesions. As the disease progressed, typical signs of upper motor neuron involvement, such as gait disturbance, extensor plantar responses, and primitive reflexes, emerged. Leukoencephalopathy, cerebral atrophy, thinning of the corpus callosum, and hypointense putamina were reported. The affected man, carrying the homozygous c.269delG; p.(Gly90Valfs*99) mutation, presented with the development of a frontal lobe syndrome at 22 years, accompanied by personality and behavioral changes. Like the first patient, he also had multiple cystic bone lesions. His bone pain started at the age of 21 and the first fracture occurred at the age of 24 (Klünemann et al. 2005).

Buthut et al. (2023) also reported p.(Ala105Argfs*84) mutation in compound heterozygosity with a novel variant c.199delC; p.(His67Thrfs*9), in a case of NHD. The c.199delC variant results in a frameshift mutation, leading to the premature termination of translation within the ECD of the protein. This causes the production of a truncated polypeptide lacking a portion of the ECD, as well as the complete transmembrane and cytoplasmic domains. During her early twenties, the patient carrying this heterozygous variant experienced a fracture in her left ankle following a horse‐riding accident. Subsequent examination by orthopedic specialists revealed the presence of bone cysts in the talus, as observed in CT scans. Additional imaging through MRI unveiled three bone cysts located within the talus and fibular malleolus, exhibiting central fat signal intensity. She also suffered from joint pain in the knees, ankles, and wrists. Following the initiation of her master's degree studies, the patient began displaying behavioral changes. She decided to withdraw from university, terminate a long‐standing relationship, relocate, and engage in high‐risk sporting activities. Her symptoms progressed to include a decline in motivation, mood swings, verbal aggression, a gradual decline in cognitive function, and impaired concentration. Complete discrete disinhibition, euphoria, Witzelsucht, logorrhoea, disturbed impulse control, increased eating, and increased distractibility were also reported (Buthut et al. 2023).

The small deletion variant, c.549del; p.(Leu184Serfs*5), identified in this study occurs within the transmembrane domain of the TREM2 protein. It is predicted to generate a truncated protein lacking a portion of the transmembrane and complete cytoplasmic domains. According to I‐mutant2.0 predictions, this substitution may decrease protein stability (DDG: −1.25). Additionally, the mutation is expected to induce nonsense‐mediated decay (NMD), a known mechanism in PLOSL2 disease. This mutation adds to a pattern observed in previously documented mutations, c.313delG; p.(Ala105Argfs84) and c.269delG; p.(Gly90Valfs99), which also leads to premature termination at codon 189, suggesting a critical functional domain beyond this point in the protein structure. This shared molecular consequence implies a pivotal role for residues beyond codon 189 in maintaining protein integrity and function.

Given that TREM2 anti‐inflammatory properties can be mediated by the C‐terminal fragment, and phagocytic activity requires the full‐length receptor (Yao et al., 2019), it is plausible to hypothesize that the deletion mutation p.(Leu184Serfs*5) resulting in a frameshift and premature termination may lead to the truncation of the TREM2 protein. This truncation would likely result in the loss of the C‐terminal fragment necessary for anti‐inflammatory functions and may impair the full functionality of the receptor required for phagocytic activity.

Furthermore, considering that the missense mutation in the transmembrane domain, c.558 G > T; p.(K186N), has been shown to disrupt signal transduction by interfering with the interaction with TYROBP, it is conceivable that the frameshift mutation resulting in a truncated protein may similarly disrupt crucial protein–protein interactions necessary for proper signaling.

In this study, consistent with previous cases, the proband exhibited a range of symptoms, including skeletal pain, joint swellings, bone cysts, frequent bone fractures, personality changes such as inappropriate and risky behaviors, apathy, bradykinesia, generalized tonic–clonic seizures, early onset dementia, and tremors. Neuroimaging revealed significant atrophy of the basal ganglia, and deep white matter periventricular hyperintensities, suggesting underlying degenerative changes. Additional neuroimaging findings included cortical atrophy, ex‐vacuo ventriculomegaly, thinning of the corpus callosum, and basal ganglia calcifications. Collectively, these neuroimaging findings provide valuable insights into the structural changes within the patient's brain.

Skeletal pain, joint swelling, bone cysts, frequent bone fractures, personality changes, early onset dementia, and tremors are common symptoms among the three affected siblings. Both brothers also experienced generalized tonic–clonic seizures and bradykinesia. The affected sister passed away at the age of 41 following a loss of mobility, highlighting the profound impact of this disorder.

## 4 CONCLUSION

In this study, we detected a novel frameshift variant causing NHD in three siblings of a consanguineous Iraqi family using WES. The c.549del; p.(Leu184Serfs*5) variant, which is predicted to lead to a complete loss of function of TREM2, can be considered as the likely pathogenic variant according to the ACMG‐AMP criteria. It is predicted to generate a truncated protein lacking a portion of the transmembrane and complete cytoplasmic domains. Null variants in *TREM2*, are predicted to cause NMD. This variant could decrease the stability of the protein, which results in protein dysfunction. It is possible to hypothesize that the c.549del; p.(Leu184Serfs*5) mutation could lead to functional impairment of TREM2, potentially compromising its anti‐inflammatory properties and phagocytic activity due to the truncation of the protein and disruption of signal transduction pathways. To our knowledge, this study reports the first case of PLOSL2 in the Iraq region. Further studies are needed to understand the implications and consequences of the identified variant on the translation and functions of the mutated TREM2 protein.

## AUTHOR CONTRIBUTIONS

M.G and N.G designed the project, N.G and M.O did the data collection, F.B. and S. Å wrote the paper, M. H. did the clinical evaluation, and all authors read and approved the final manuscript.

## FUNDING INFORMATION

No specific funding has been provided for research.

## CONFLICT OF INTEREST STATEMENT

The authors declare no competing interests.

## ETHICS STATEMENT

The written, informed consent was obtained from all participants or their respective guardians. The authors declare that they have conducted the project ethically in accordance with the World Medical Association Declaration of Helsinki. The study was approved by the local medical ethics committee of DeNA laboratory, Tehran, Iran.

## Supporting information


Table S1:

## Data Availability

The data that support the findings of this study are available from the corresponding author upon reasonable request.
