# Supplementary material for: Homozygous TREM2 c.549del; p.(Leu184Serfs*5) variant causing Nasu‐Hakola disease in three siblings in a consanguineous Iraqi family: Case report and review of literature
Source: Mol Genet Genomic Med. 2024 Jun 18;12(6):e2476. doi: 10.1002/mgg3.2476 (PMC11184573; doi:10.1002/mgg3.2476)
Supplement: Supplementary file 1 — Table S1: [file MGG3-12-e2476-s001.docx]

| **Variant** | **Ethnicity** | **Family history** | **Gender** | **Skeletal pain** | **Bone problems** | **Personality and behavioral changes** | **Percentile Demetia** | **Brain abnormalities** | **Ganglia calcification** | **Diffuse slowing in the electroencephalogram** | **Agnostic-aphasic-apraxic symptomes** | **Primitive reflexes** | **Convulsion** | **Epileptic seizure** | **Upper motor neuron involvement** | **Urinary retention** | **Other symptoms** | **Ref** |
| --- | --- | --- | --- | --- | --- | --- | --- | --- | --- | --- | --- | --- | --- | --- | --- | --- | --- | --- |
| **c. 233G>A; p.(****Trp78Stop) ^#^** | **Swedish** | **NS** | **NS** | **+** | **Bone cysts and/or fractures** | **Frontal lobe syndrome; euphoria and loss of social inhibitions** | **+** | **Brain atrophy** | **NS** | **-** | **+** | **+** | **-** | **NS** | **NS** | **NS** | **NS** | **Paloneva et al., 2002** |
| **c.558 G>T; p.(Lys186Asn)** | **Norwegian** | **Siblings** | **F** | **+** |  |  | **+** |  | **NS** | **NS** | **+** | **NS** | **NS** | **NS** | **NS** | **NS** | **NS** |  |
|  |  |  | **F** | **+** |  |  | **+** |  | **NS** | **-** | **NS** | **NS** | **+** | **NS** | **NS** | **NS** | **NS** |  |
| **c.401A>G; p.(Asp134Gly)** | **American (Slovakian)** | **NS** | **NS** | **+** |  |  | **+** |  | **NS** | **+** | **-** | **+** | **+** | **NS** | **NS** | **NS** | **NS** |  |
| **c.132G>A; p.(Trp44Stop)** | **Bolivian** | **NS** | **NS** | **+** |  |  | **+** |  | **NS** | **+** | **+** | **+** | **+Following neurosurgery** | **NS** | **NS** | **NS** | **NS** |  |
| **c.482+2T>C** | **Italian** | **Siblings** | **F** | **+** |  |  | **+** |  | **NS** | **+** | **+** | **-** | **-** | **NS** | **NS** | **NS** | **NS** |  |
|  |  |  | **F** | **-** |  |  | **+** |  | **NS** | **-** | **+** | **+** | **+** | **NS** | **NS** | **NS** | **NS** |  |
|  | **Japanese** | **Siblings** | **F** | **NS** | **Multifocal radiolucent lesions** | **Personality changes; laziness, decreased ability to concentrate, a lack of insight, poor judgment, social inhibitions, apathy, and memory disturbances** | **+** | **Moderate hypoperfusion in frontotemporal cortex with preservation of the cerebral blood flow in the basal ganglia, moderate atrophy and marked dilatation of the lateral ventricle** | **+** | **+** | **NS** | **NS** | **NS** | **+** | **NS** | **NS** | **NS** | **Numasawa et al., 2011** |
|  |  |  | **M** | **NS** |  | **NS** | **+** | **NS** | **NS** | **NS** | **NS** | **NS** | **NS** | **+** | **NS** | **NS** | **NS** |  |
|  |  |  | **M** | **NS** |  | **NS** | **+** | **NS** | **NS** | **NS** | **NS** | **NS** | **NS** | **-** | **NS** | **NS** | **NS** |  |
| **c.40G>T; p.(Glu14stop)** | **German** | **NS** | **NS** | **NS** | **Bone cysts and/or fractures** | **NS** | **+** | **NS** | **NS** | **NS** | **NS** | **NS** | **NS** | **NS** | **NS** | **NS** | **NS** | **Paloneva et al., 2003** |
| **c.97C>T; p.(Gln33stop)** | **Italian** | **Siblings** | **F** | **+** | **Bone fractures** | **Insidious personality changes; loss of social inhibition and judgment, depression with suicidal ideation, and frontal signs** | **+** | **Diffuse cerebral atrophy** | **+** | **NS** | **NS** | **+** | **NS** | **+** | **Mild Apraxia** | **NS** | **Dyscalculia**  **Disorientation**  **progressive cognitive function deterioration**  **Cerebral ictal events**  **Vegetative state due to disease progression** | **Soragna et al., 2003** |
|  |  |  | **F** | **+** | **Cystic bone lesions** | **Frontal dysfunction; progressive loss of judgment, depression, personality changes, and uninhibited attitudes** | **+** | **Sever diffuse cerebral atrophy** | **+** | **NS** | **NS** | **NS** | **NS** | **NS** | **NS** | **NS** | **Dyscalculia**  **Dysgraphia**  **Cognitive function deterioration** |  |
|  | **Belgian** | **Siblings** | **M** | **NS** | **Multiple cystic bone lesions and fractures** | **Frontal lobe syndrome: personality and behavioral changes** | **+** | **Cerebral atrophy, leukoencephalopathy, thinning of the corpus callosum, caudate atrophy, and hypointense putamina** | **+** | **NS** | **NS** | **NS** | **NS** | **NS** | **NS** | **NS** | **NS** | **Klünemann et al., 2005** |
|  |  |  | **F** | **NS** |  |  | **+** | **Cerebral atrophy, leukoencephalopathy, thinning of the corpus callosum, caudate atrophy, hypointense thalamus, and hypointense putamina** | **+** | **NS** | **NS** | **NS** | **NS** | **NS** | **NS** | **NS** | **NS** |  |
|  | **NA** | **NS** | **F** | **NS** | **Multiple asymptomatic cystic bone lesions of hands and feet** | **Personality changes, social inhibition, and memory disturbances** | **+** | **Diffuse cortical atrophy, white-matter loss, and cortical hypometabolism** | **NS** | **NS** | **NS** | **NS** | **NS** | **NS** | **NS** | **+** | **Disorientation**  **Complete dependence on daily activities** | **Ghezzi et al., 2017** |
| **313delG;**  **p.(Ala105Argfs*84)** | **German** | **NS** | **F** | **-** | **Multiple cystic bone lesions** | **Frontal lobe syndrome: personality and behavioral changes** | **+** | **Leukoencephalopathy, cerebral atrophy, thinning of corpus callosum, and hypointense putamina** | **+** | **NS** | **NS** | **+** | **NS** | **NS** | **Gait disturbance and extensor plantar responses** | **NS** | **NS** | **Klünemann et al., 2005** |
| **269DelG;**  **p.(Gly90Valfs*9)** | **France (Turkey)** | **NS** | **M** | **+** | **Multiple cystic bone lesions and fractures** | **Frontal lobe syndrome; personality and behavioral changes** | **+** | **Leukoencephalopathy, cerebral atrophy, hypointense thalamus and hypointense putamina** | **+** | **NS** | **NS** | **NS** | **NS** | **NS** | **NS** | **NS** | **NS** |  |
| **c377T>G; p.(Val126Gly)** | **Canada** | **NS** | **F** | **-** | **Multiple cystic bone lesions, and 2 fractures** | **Frontal lobe syndrome; personality and behavioral changes** | **+** | **Leukoencephalopathy, cerebral atrophy, thinning of corpus callosum, and hypointense thalamus** | **NS** | **NS** | **NS** | **NS** | **NS** | **NS** | **NS** | **NS** | **NS** |  |
|  | **UK (Sri Lanka)** | **NS** | **M** | **+** | **Multiple cystic bone lesions, and Fractures** | **Frontal lobe syndrome; personality and behavioral changes** | **+** | **Cerebral atrophy, leukoencephalopathy, thinning of the corpus callosum, caudate atrophy, hypointense thalamus and hypointense putamina** | **NS** | **NS** | **NS** | **NS** | **NS** | **NS** | **NS** | **NS** | **NS** |  |
| **Variant** | **Ethnicity** | **Family history** | **Gender** | **Skeletal pain** | **Bone problems** | **Personality and behavioral changes** | **Percentile Demetia** | **Brain abnormalities** | **Ganglia calcification** | **Diffuse slowing in the electroencephalogram** | **Agnostic-aphasic-apraxic symptomes** | **Primitive reflexes** | **Convulsion** | **Epileptic seizure** | **Upper motor neuron involvement** | **Urinary retention** | **Other symptoms** | **Ref** |
| **c.113A>G; p.(Tyr38Cys)** | **NA** | **Nephew-uncle** | **F** | **+** | **Osteoporotic, cystic lesions and repetitive fractures** | **Personality, and behavior changes** | **+** | **Cerebral atrophy, diffuse signal changes in white matter with global brain atrophy, thin corpus callosum, and bilateral hypometabolic pattern** | **+mild** | **NS** | **NS** | **NS** | **NS** | **NS** | **NS** | **NS** | **Cognitive deficits** | **Köseoğlu et al., 2018** |
|  |  |  | **M** | **NS** | **Osteoporotic, cystic lesions and repetitive fractures** | **Personality and behavioral changes** | **+** | **Cerebral atrophy, diffuse signal alterations in white matter, global brain atrophy, a diffusely thinned corpus callosum, reduced anterior cingulate gyrus volume and bilateral hypometabolism** | **+mild** | **NS** | **NS** | **NS** | **NS** | **NS** | **NS** | **NS** | **NS** |  |
| **c.150G > T; p.(Trp50Cys)** | **Greek** | **NS** | **F** | **NS** | **Multiple diffuse cystic, osteosclerotic lesions and fractures** | **Frontal lobe dysfunction; personality, and behavioral alterations, emotional instability, euphoria, lack of insight, bulimia, perseverations, stereotyped behaviors, and increased speech velocity** | **+** | **Cerebellar atrophy, thin corpus callosum, diffuse symmetrical hyperintensities in the white matter** | **+** | **NS** | **NS** | **NS** | **NS** | **NS** | **Gait instability** | **+** | **progressive cognitive decline**  **Episodes of acute, short-lasting, neuralgic pain in the right occipital area at the age of 17** | **Dardiotis et al., 2017** |
| **c.197C > T; p.(Thr66Met)** | **Japanese** | **Her sister had died of NHD** | **NS** | **NS** | **Bone fractures and lipomembranous lesions of the bone** | **Increased Speech velocity** | **+** | **Severe brain atrophy with diffuse loss of myelin and axons in the white matter** | **NS** | **NS** | **NS** | **NS** | **NS** | **+** | **Extrapyramidal signs,**  **gait instability**  **gait disturbance**  **extensor plantar responses** | **NS** | **Passed away at 41** | **Sasaki et al. 2015** |
| **c.257A>T; p.(Asp86Val)** | **NS** | **NS** | **NS** | **NS** | **Bone cysts without any fractures in one patient**  **History of fractures but no bone cysts in one patient**  **Both bone cysts and a history of fractures in one patient** | **Behavioral changes with cognitive decline** | **+** | **Thinning of the Corpus callosum, White matter involvement, and Symmetrical global atrophy of the brain** | **4 out of 5 patients** | **NS** | **NS** | **NS** | **NS** | **3 out of 5 patients** | **Parkinsonism** | **NS** | **NS** | **Samanci et al. 2021** |
|  | **NS** | **NS** | **NS** | **NS** |  |  | **+** |  |  | **NS** | **NS** | **NS** | **NS** |  |  | **NS** | **NS** |  |
|  | **NS** | **NS** | **NS** | **NS** |  |  | **+** |  |  | **NS** | **NS** | **NS** | **NS** |  |  | **NS** | **NS** |  |
|  | **NS** | **NS** | **NS** | **NS** |  |  | **+** |  |  | **NS** | **NS** | **NS** | **NS** |  |  | **NS** | **NS** |  |
|  | **NS** | **NS** | **NS** | **NS** |  |  | **+** |  |  | **NS** | **NS** | **NS** | **NS** |  |  | **NS** | **NS** |  |
| **c.391+1G>A** | **Chinese** | **NS** | **F** | **NS** | **Mild radiological bone involvement, possible cystic swelling of the metaphyses of the long bones in the carpal and talus bones** | **Frontal lobe syndrome; behavioral changes, loss of motivation, an inability to concentrate, poor judgments, neglect of personal hygiene, disinhibition, an unusually increased appetite, moderate aggression, and indifference to family members and social life.**  **, and memory loss** | **+** | **Moderate cortical atrophy, ventricular enlargement, thinning of the corpus callosum, and notable diffuse hyperintensities in the periventricular, frontal, temporal, and occipitoparietal white matter.** | **NS** | **NS** | **NS** | **Brisk** | **NS** | **NS** | **Bradykinesia**  **Mild Ataxia**  **Positive bilateral Hoffman, Babinski, and Chaddock signs**  **Parkinsonism** | **+** | **Cognitive impairment** | **Le et al. 2014** |
| **c.199delC; p.(His67Thrfs*9) *** |  |  | **F** | **+** | **a fracture history in her left ankle following a horse-riding accident, three bone cysts located within the talus and fibular malleolus, and Joint pain in the knees, ankles, and wrists.** | **Behavioral changes, a decline in motivation, mood swings, verbal aggression, a gradual decline in cognitive function** | **+** | **MRI imaging of the brain revealed signs of supratentorial cortical and subcortical atrophy, along with mild periventricular leukoencephalopathy, and relatively preserved infratentorial structures** | **+** | **No signs of epileptiform abnormalities in the EEG, an asymmetric frontotemporal dysrhythmia during rest, and an increase in delta and beta power.** | **NS** | **-** | **NS** | **NS** | **-** | **NS** | **Cognitive impairment** | **Buthut et al., 2023** |
| **c.549delA; p.(Leu184Serfs*5)** | **Iraqi** | **M** | **Siblings** | **+** | **joint swellings, bone cysts, frequent bone fractures** | **personality changes such as inappropriate and risky behaviors, and apathy** | **+** | **atrophy of the basal ganglia, deep white matter periventricular hyperintensities. cortical atrophy, ex-vacuo ventriculomegaly, thinning of the corpus callosum.** | **+** | **-** | **-** | **-** | **-** | **+** | **Bradykinesia**  **Tremors** | **-** | **NS** | **This study** |
|  |  | **M** |  | **+** |  | **personality changes involving poor judgment** | **+** | **NS** | **NS** | **-** | **-** | **-** | **-** | **+** | **Bradykinesia**  **Tremors** | **-** | **NS** |  |
|  |  | **F** |  | **+** |  | **personality changes** | **+** | **NS** | **NS** | **-** | **-** | **-** | **-** | **NS** | **Tremors** | **-** | **passed away at the age of 41** |  |

**# The same mutation was also detected in another Swedish family with three affected individuals, NS: Not stated, * This variant is identified in compound heterozygosity with c.313delG;p.Ala105Argfs*84**
